# The regulatory landscape of biosimilars: WHO efforts and progress made from 2009 to 2019

**DOI:** 10.1016/j.biologicals.2020.02.005

**Published:** 2020-05

**Authors:** Hye-Na Kang, Robin Thorpe, Ivana Knezevic, Survey participants from 19 countries, Carolina Damas Rocha Zarate Blades, Mary Casas Levano, Jing Yin Chew, Mumbi Bernice Chilufya, Parichard Chirachanakul, Hui Ming Chua, Ali Vasheghani Farahani, Mariam Raouf Wefky Ghobrial, Suna Habahbeh, Hugo Hamel, Gi Hyun Kim, Violeta Perez Rodriguez, Desi Eka Putri, Jacqueline Rodgers, Maria Savkina, Oleh Semeniuk, Shraddha Srivastava, Meenu Wadhwa, Teruhide Yamaguchi

**Affiliations:** c Brazilian Health Regulatory Agency (ANVISA), Brasilia, Brazil; d General Directorate of Medicines Supplies and Drugs (DIGEMID), San Miguel, Peru; e Health Sciences Authority, Singapore; f Zambia Medicines Regulatory Authority, Lusaka, Zambia; g Food and Drug Administration, Nonthaburi, Thailand; h National Pharmaceutical Regulatory Agency, Selangor, Malaysia; i Iran Food and Drug Administration, Tehran, Iran; j Central Administration for Pharmaceutical Affairs, Cairo, Egypt; k Jordan Food and Drug Administration, Amman, Jordan; l Health Canada, Ottawa, Canada; m Ministry of Food and Drug Safety, Osong, Republic of Korea; n Centro para El Control Estatal de Medicamentos, Equipos y Dispositivos Médicos (CECMED), Habana, Cuba; o Badan POM (National Agency of Drug and Food Control), Jakarta, Indonesia; p Food and Drugs Authority, Accra, Ghana; q The FSBI “SCEEMP of the Ministry of Health of the Russian Federation, Moscow, Russian Federation; r Ministry of Health of Ukraine, Kyiv, Ukraine; s Central Drug Standards Control Organization (CDSCO), Ministry of Health & Family Welfare, New Delhi, India; t National Institute for Biological Standards and Control, Medicines and Healthcare Products Regulatory Agency, Potters Bar, United Kingdom; u Pharmaceuticals and Medical Devices Agency and Kanazawa Institute of Technology, Tokyo, Japan; a World Health Organization, Department of Health Products Policy and Standards, Avenue Appia 20, CH-1211, Geneva, Switzerland; b Independent Expert, Welwyn, United Kingdom

**Keywords:** Biosimilar, Similar biotherapeutic product, Regulatory guidelines, Survey, WHO

## Abstract

The World Health Assembly in 2014 adopted a resolution that mandates both Member States and the WHO Secretariat to facilitate access to biotherapeutic products in a way that ensures their quality, safety and efficacy. The availability of biosimilars is expected to increase access to biotherapeutic products by providing more treatment options triggering competition which would lead to a consistent reduction in the average price of treatment. Since the WHO guidelines for regulatory evaluation of biosimilars were issued in 2009, WHO has provided immense effort towards harmonizing the terminology and the regulatory framework for biosimilars globally. This article describes the progress made and the regulatory landscape changes for biosimilars in 21 countries during the past ten years. Based on the information from regulators and from publicly available data, the following has been identified: 1) WHO guidelines have contributed to setting the regulatory framework for biosimilars in countries and increasing regulatory convergence at global level; 2) terminology used for biosimilars is more consistent than in the past; 3) biosimilars are now approved in all participating countries; and 4) the dominant product class for candidate biosimilars under development is monoclonal antibodies.

## 1 Introduction

The World Health Organization (WHO) is not a regulatory authority, but it has a clear mandate to support regulatory authorities in its 194 Member States. More precisely, one of the WHO core functions is “setting norms and standards and promoting and monitoring their implementation”. The WHO Mission in the context of the regulation of biologicals is to provide documents with globally agreed principles and experts’ advice that serve as a basis for establishing or updating national regulatory requirements. WHO guidelines and recommendations for vaccines and other biologicals are considered as WHO written standards and they also serve as a basis for WHO prequalification.

The WHO guidelines on the evaluation of similar biotherapeutic products (SBPs; hereafter referred to as ‘the Guidelines’) [1] were developed to provide a globally acceptable set of basic principles for licensing biosimilars and to serve as a basis for setting national licensing requirements. Since the adoption of the WHO Guidelines by the Expert Committee on Biological Standardization (ECBS) in 2009, several WHO implementation workshops have been held to discuss the WHO Guidelines with regulators and manufacturers from more than 60 countries. Regulators in WHO Member States are playing a pivotal role in implementing WHO guiding principles in their national regulations. WHO is facilitating that process by organizing implementation workshops with lectures, case studies and review of examples of product approvals which serve as opportunities to discuss scientific but also practical aspects in the forum of regulators, manufacturers and academia. The key lectures, outcomes of the discussions and reports from countries have been published including very useful case studies [[2], [3], [4], [5], [6], [7], [8], [9]].

Prior to the workshops, in most cases, WHO conducted a survey to capture the status of national requirements related to the regulatory evaluation of such products with particular emphasis on whether or not the current WHO Guidelines had been, or were to be, incorporated into national requirements.

Towards WHO efforts on biotherapeutics, WHO developed the WHO guidelines on post-approval changes to biotherapeutic products which were adopted by the WHO ECBS in 2017 [10]. Since the need for promoting and assisting Member States in implementation of WHO standards has been clearly identified, the first implementation workshop for these guidelines was planned to take place from 25 to 26 June 2019 in Seoul, Republic of Korea. As a part of the preparation for the workshop, a survey was conducted among the 20 workshop participating countries to review the current situation on regulation and approval of biotherapeutic products and SBPs (also called biosimilars) as well as summarize any challenges encountered. The experience with the survey conducted previously, in 2010 was that many countries and regions had made progress in developing a regulatory framework for biotherapeutic products including SBP. Nevertheless, it also revealed problems with inappropriate application of the principles outlined in the 2009 WHO Guidelines [11].

As described above, WHO has provided considerable effort and assistance to regulatory authorities in implementing the principles of evaluation included in the guidelines into regulatory practice. One example of these efforts is the recent publication of a Q&A document to complement the WHO Guidelines for biosimilars [1,12]. The questions in the document were selected on the basis of those frequently asked by regulators during implementation workshops on the 2009 WHO Guidelines conducted during the past nine years. The expectation of WHO is that the Q&A document will provide scientific and regulatory update and clarity for the users of WHO Guidelines.

From the survey conducted in 2019, WHO aims to update the information on the global situation and identify areas where further support to its Member States needs to be provided. In this article, the information accrued on regulation and approval of SBPs in the countries participating in the survey are presented and discussed. The findings on challenges and future opportunities will be published in a separate article in the near future.

## 2 Methodology

For the survey, a questionnaire was prepared by WHO in the form of a template for completion by participating regulatory authorities. The template was similar to that used for the previous (August 2010) WHO survey [11] but updated to include additional data such as classification of insulin and low molecular weight heparin (LMWH), indication of guidelines adopted and intention to develop the national guidelines, and existence of non-innovator or me-too products.

The representatives of 20 countries for the above-mentioned workshop in June 2019 nominated by the WHO region offices or countries were invited for the survey. It included participants from Brazil, Canada, China, Cuba, Egypt, Ghana, India, Indonesia, Iran, Japan, Jordan, Malaysia, Peru, Republic of Korea, Russia, Singapore, Thailand, Ukraine, UK and Zambia who were involved in WHO implementation activities in the past ten years. The feedback from UK refers to the situation in the EU rather than specifically for the UK.

Information was assessed from a total of 21 countries. This comprised the 20 participants in the survey and the USA. Information from the USA was not derived from the survey, but from publicly available data, which has been published and is comprehensive.

The data received were assessed and analysed at WHO according to detailed analysis as described below in the results section and figures/tables. The assessment results presented in this article are based on the information and data provided by survey participants from 20 countries in May–June 2019 and updated and confirmed in August 2019. It is also important to note that biosimilars approved in certain countries might not have been approved following a strict regulatory process as recommended by the WHO Guidelines.

## 3 Results

### 3.1 Regulatory guidelines

The Committee for Medical Products for Human Use (CHMP) of the EU was the first regulatory agency to draft guidance documents for biosimilars. To date, a number of guidelines have been adopted by the EU including those providing general guidance, more specific guidance on quality issues, non-clinical and clinical aspects and numerous guidelines on particular products [13]. Furthermore, guidelines on immunogenicity assessment which includes assessment of biosimilars have also been elaborated. Most of these have been revised to take account of experience gained with the development and approval of biosimilars and changes required by advances in science, technology and clinical practice. Such revision is undertaken only when necessary and not simply due to the age of the guideline.

**Fig. 1:**
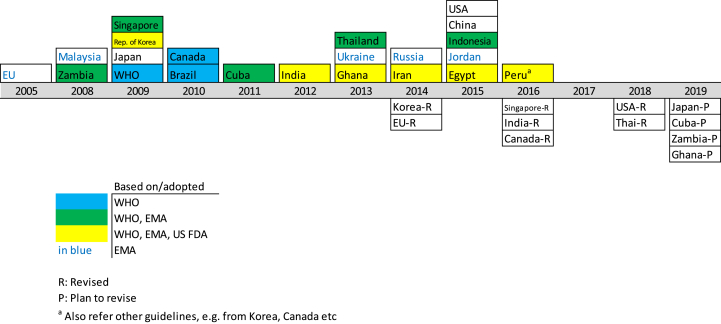
Years when guidelines were made available by WHO and 21 regulatory authorities.

Since the adoption of the EU guidance, many other regulatory authorities have produced/adopted biosimilar guidelines (see Fig. 1). Many authorities have based their guidelines on existing guidelines produced by other agencies mainly by WHO, but also by EU and USFDA. The extent of overlap of the guidelines varies.

Considerable progress in adoption of guidelines has been made since the first survey was made in 2010 (see Fig. 1). At this time, in addition to the EU, a number of countries had guidelines in place - Canada, Japan, Malaysia, Republic of Korea, Singapore while others - Brazil, Cuba and Thailand were developing such guidance. Data shows that most countries had guidelines in place by 2015 and, as can be seen, all participating countries now have relevant guidelines adopted which contrasts with the situation 9 years ago. In particular, China and India now have guidelines which was not the case previously and, in 2010, did not even have any plans for the preparation of such guidelines [11].

In most cases, adoption of guidelines is complemented by the establishment and publication of regulatory procedures for the assessment and approval of biosimilars.

During the period between the two surveys (August 2010 and August 2019), several countries have revised their guidelines. There are various reasons for this, including changes in nomenclature and terminology used for biosimilars and their assessment, changes in source and other aspects of the reference product, changes in clinical reqirements, changes in legal aspects relating to biosimilars, changes in labelling requirements and changes for improving clarity of the guideline. All regulatory guidelines are considered ‘living documents’ that need to be updated in line with advances in scientific knowledge and experience.

### 3.2 Terminology/nomenclature

Terminology used for biosimilars has been inconsistent in the past [11]. The same term has sometimes been used for different types of product and vice-versa. This has caused some confusion. What is understood by particular terms is also inconsistent, even including the term ‘biosimilar’ itself.

As noted previously [11], some countries have approved non-innovator products. They are not biosimilars as defined in the WHO, EU or USA biosimilar guidelines (and most other biosimilar guidance generally available) which require head-to-head comparison of a biosimilar candidate with a licensed originator product with the goal of establishing similarity in quality, safety and efficacy. Such products are mainly copies of innovator products that are licensed in various countries and may have a long and established history of good efficacy and safety. They do not contain a new biological entity. Partial comparative studies at the quality level have been performed for the purpose of licensing of these products, and a comparison of clinical data for the products with published (literature) data for originator products has been accepted for licensure of these non-innovator products. However, it is often unclear whether these products are called ‘biosimilars’ or if alternative terminology is used. Some countries have a regulatory process for these products which involves an abbreviated pathway to licensure compared to innovator products. Other countries do not have such a process, e.g. Canada, EU, USA.

However, there has been some progress made since the first survey in converging on consensus use of nomenclature although this is far from comprehensive or complete. It seems that there is a trend towards adopting the term ‘biosimilar’ for products which have been subjected to the rigorous comparability exercise with the reference product as defined in WHO, EU and USFDA guidelines, but this is not yet universal. Canada, for example has abandoned the use of the term ‘subsequent entry biologic’ used in 2010 and moved to ‘biosimilar’ in 2016 (see Table 1). This trend is welcome as it avoids confusion, moves towards consistency with guidelines and harmonisation of terms used for the same products and avoids confusion with subsequent entry biologics which are not biosimilars, like non-innovator products.

**Table 1:**
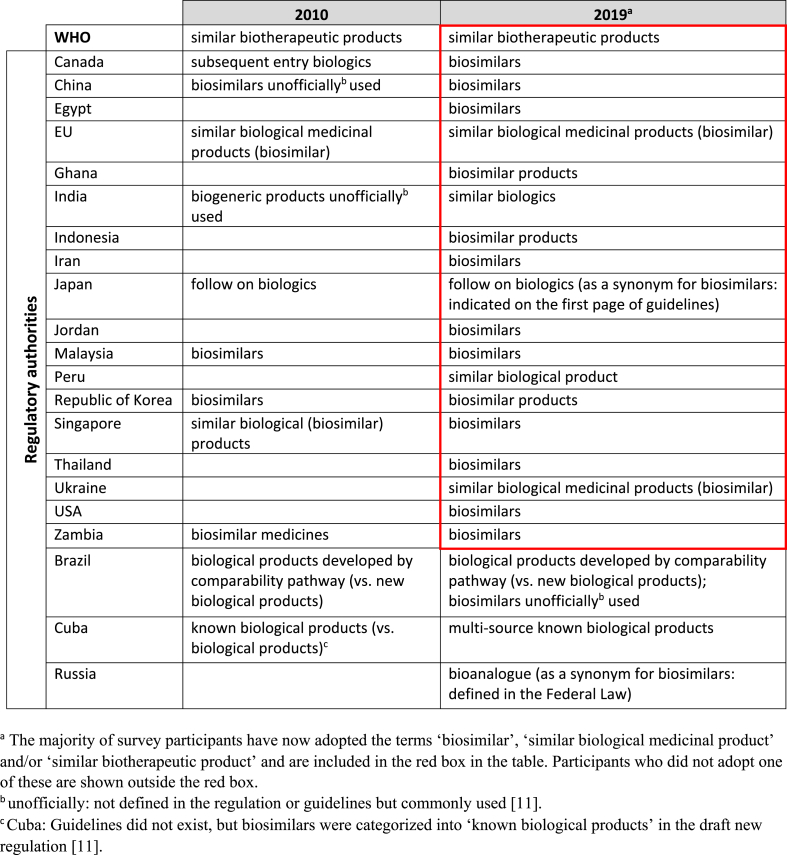
Terminologies used in regulatory guidelines

The terms used during the 2010 and 2019 surveys are summarized in Table 1. WHO still uses the original ‘similar biotherapeutic product’ term as was the case in 2010 to clarify the scope of this term in the context of WHO Guidelines which do not apply to all biologicals but only to biotherapeutic products. In general, it seems that the terms ‘biosimilar’, ‘similar biological medicinal product’ and ‘similar biotherapeutic products’ are used and refer to the same type of product (that defined in the WHO, EU and USFDA biosimilar guidelines). Other terms still in use such as ‘bioanalogue’ and ‘multi source known biological products’ do not in fact refer to biosimilars, but to non-innovator products which are not biosimilars. Brazil uses a unique name ‘biological products developed by comparability pathway’ for biosimilars which distinguishes these products from new products or those approved without comparability assessment; the term biosimilar is unofficially used for these products. Similar situations may exist elsewhere although the nomenclature used may be different.

The term ‘biogeneric’ which was in use at the time of the first survey, seems to have been largely abandoned. The term ‘generic’ is not appropriate and is no longer in use for any type of biological.

### 3.3 Approval of biosimilars

The first biosimilar, Omnitrope which was a somatropin product, was approved in the EU in 2006. This was followed by a series of approvals of other biosimilars in the EU. As of August 2019, there are 61 approved biosimilars in the EU and all have safety and efficacy profiles comparable to their innovator reference products (data for this derived from many years of post-marketing surveillance). In some cases, the same substance has been given different brand names or has been approved by more than one marketing authorization holder (largely for commercialization and marketing region allocation). A few products have been withdrawn or rejected and the reasons for these decisions have not always been made publicly available, but no products have been withdrawn because of safety or efficacy problems.

Following the EU's lead in this area, other countries have approved biosimilars and this trend has increased throughout the survey period, with more countries and more products being involved. In some cases, availability of guidelines and adoption of regulatory approval processes have been rapidly followed by approval of biosimilars. In many cases the same biosimilar substance is approved in several or even many countries, sometimes, but not always with same name.

The current (August 2019) situation with approved biosimilars is shown in Table 2.

**Table 2:** Numbers of biosimilars approved by regulatory authorities in 21 countries (August 2019).

	Brazil	Canada	China^b^	Cuba	Egypt	EU^c^	Ghana	India	Indonesia^d^	Iran	Japan	Jordan	Malaysia	Peru	Republic of Korea	Russia	Singapore	Thailand	Ukraine	USA	Zambia	Under development
Others^a^								14		1												7
hCG								2														
Teriparatide						2		3														1
Somatropin	1	1		1	1	2		1			1	1	1		1		1		1			2
LMWH	1					2																3
Peg-interferon								2		1												1
Interferon							1	6		6						6						1
Human insulin	2						7	4	6	1		1	1			10					1	
Insulin glargine	2					3		2	3		2		1		2	1	1					3
Insulin lispro	1	1				1										1						
FSH						2	2	5		1									1			1
Peg-filgrastim		2				6		7		1										2		16
Filgrastim	2	1			2	9		9	4	2	5	2	2			4	2	4	2	2		9
Etanercept	1	2				2		2		1	1				3					2		16
EPO						5	2	11	5	2	1	1	3			3			1	1	3	2
Darbepoetin								4			2				1							2
Rituximab	2	1	1	2		6		5	2	2	1	2	1	2	1	1	1	3	1	2		23
Infliximab	3	3				4		1			4	1	1	1	2	2	1			3		6
Cetuximab																						6
Trastuzumab	4	1		2	1	5	1	5	3	1	4	1	3	1	2	1		4	1	5	2	20
Ranibizumab								1														8
Pertuzumab																						5
Bevacizumab	1	1		1		2		5		1	1		1			1		1		2	2	25
Adalimumab	1	2				10		4		1		1	1		1	1	1	1		4		20
total	21	15	1	6	4	61	13	93	23	21	22	10	15	4	13	31	7	13	7	23	8	177

hCG: Human chorionic gonadotropin; LMWH: Low Molecular Weight Heparin; FSH: Follicle stimulating hormone; EPO: Erythropoietin.

a Others: India (abciximab, becaplermin, denosumab, granulocyte macrophage colony, hepatitis B vaccine, itolizumab, molgramostim, raburicase, reteplase, streptokinase); Iran (eptacog alfa); Under development (aflibercept, basiliximab, certolizumab, eculizumab, liraglutide, palivizumab).

b China: Last update in June 2019.

c EU: 7 biosimilars approved then withdrawn (2 adalimumab, 2 filgrastim, 1 insulin glargine, 1 rituximab, 1 somatropin).

d Indonesia: 3 biosimilars approved then withdrawn (3 human insulins).

A synopsis of the situation with approved biosimilars according to regulators of countries which participated in the survey is as follows:

Brazil-has approved 21 biosimilars all since 2010. Several monoclonal antibodies (mAbs) are approved including 4 trastuzumab products.

Canada-has approved 15 biosimilars; one (somatropin) was approved in 2009, all the rest since 2010. This includes several mAbs including 3 infliximabs.

China-has approved 1 biosimilar (rituximab) in 2019.

Cuba-has approved 6 biosimilars including 1 somatropin (the first approved biosimilar in 2014) and 5 mAbs (3 of them produced in Russia).

Egypt-has 4 approved biosimilar products including 2 filgrastims. The first was approved in 2016. More recently 1 mAb product has been approved (trastuzumab).

EU- has the largest number of approved biosimilars (61) which comprise many classes of products including somatropin, erythropoietin, filgrastim, pegfilgrastim, heparin, follitropin, insulin, etanercept, teriparatide and several mAbs. This compares with only 14 biosimilars approved in 2010. A few applications for biosimilars have been refused and 7 approved products have been withdrawn.

Ghana-has approved 13 biosimilars during the survey period; none were approved before 2010. These include 7 insulins and 1 mAb produced in India.

India-has approved more than 90 biosimilars, the first being approved in 2001 (erythropoietin). These include those product classes approved in EU, but also human chorionic gonadotrophin hormone, darbepoietin, molgramostim, interferons alfa and beta, many insulins and insulin derivatives, peg-interferon alfa, tenecteplase, rasburicase, hepatitis B vaccine, and several mAbs.

Indonesia-has 23 approved biosimilars, the first being approved in 2001 (erythropoietin). These include many insulins, several erythropoietins, filgrastims and 5 mAbs.

Iran-has 21 approved biosimilars the first being approved in 1998 (interferon alfa 2b and erythropoietin). More recently, mAb biosimilars have been approved.

Japan-has approved 22 biosimilars, the first being somatropin in 2009. These include several filgrastims and mAbs.

Jordan-has 10 approved biosimilars, the first (erythropoietin) being approved in 2012. A number of mAb biosimilars are included.

Malaysia-has approved 15 biosimilars the first being somatropin in 2010. These include filgrastims, erythropoietins and mAbs.

Peru-has approved 4 biosimilars starting with infliximab in 2017. More recently rituximab and trastuzumab biosimilars have been approved.

Republic of Korea-has approved 13 biosimilars starting with infliximab in 2012. This was quickly followed by biosimilar trastuzumab and a range of other substances.

Russia-has 31 approved biosimilars the first being approved in 2010. These include filgrastims, erythropoietin, interferon alfa-2b, interferon beta, many insulins, and some mAbs.

Singapore-has approved 7 biosimilars starting with somatropin in 2009 and now includes biosimilar mAbs.

Thailand-has approved 13 biosimilars since 2017.

Ukraine-has 7 approved biosimilars, the first (filgrastim and erythropoietin) approved in 2012. Recently mAb biosimilars have been approved.

USA-approved filgrastim as the first biosimilar product in 2015. Since then a total of 23 biosimilars have been approved. Three insulin products (2 glargine and 1 lispro) were approved as follow on products (not officially biosimilars) due to administrative reasons. As well as having a pathway for approval of biosimilars (not interchangeable) the US has a process for approving interchangeable products, but none of these have been approved to date.

Zambia-has approved 8 biosimilars including recently approved mAbs. This compares with only 1 erythropoietin biosimilar approved before the start of the survey period (2010).

Biosimilars are now approved in all countries participating in the survey, which was not the case at the time of the previous survey. Several applications for licenses/authorisations for biosimilar products have been turned down by some countries.

The source/manufacture of biosimilars is very varied although many are made by companies with a strong commitment to producing biosimilars. Many are produced by ‘big pharma’ or satellite companies owned by big pharma, e.g. in the EU and USA. Several are locally produced and there are some relatively small biosimilar manufacturers. Some biosimilar producers are also producers of chemical generics. In some cases, no biosimilars are produced locally, e.g. for Ghana and Zambia most are produced in India. In Iran, all are from local (Iranian) manufacturers and in India most are from local Indian manufacturers. The one biosimilar approved in China is locally produced.

**Fig. 2:**
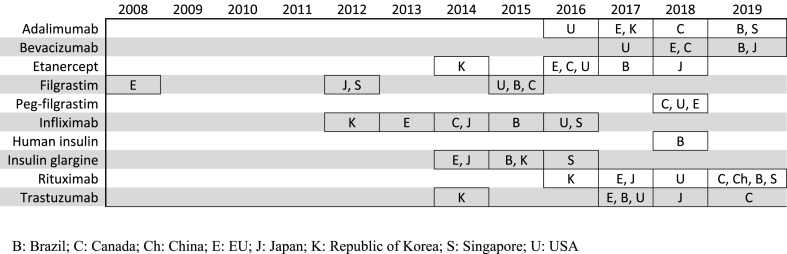
First biosimilar availability of selected major products in some participating countries.

Some biosimilars are major products or even ‘blockbusters’ (see Table 3) and Fig. 2 shows the pattern of approval of some such biosimilars in different countries that took part in the survey (Brazil, Canada, China, EU, Japan, Republic of Korea, Singapore, USA). Many of these blockbusters are mAbs and this product class now makes up a significant proportion of the biosimilars approved by many countries.

**Table 3:** Blockbuster^a^ products in the USA [14].

Brand name	Indication	Global sales in 2017	International Nonproprietary Name
Humira	Rheumatoid arthritis, plaque psoriasis, Crohn's disease, ulcerative colitis, ankylosing spondylitis, psoriatic arthritis, polyarticular juvenile idiopathic arthritis	$18.4 billion	Adalimumab
Rituxan	Non-Hodgkins lymphoma, chronic lymphocytic leukaemia, rheumatoid arthritis	$9.2 billion	Rituximab
Enbrel	Rheumatoid arthritis, plaque psoriasis, psoriatic arthritis	$7.9 billion	Etanercept
Herceptin	HER2+ breast cancer	$7.4 billion	Trastuzumab
Avastin	Breast, colorectal, kidney, non-small-cell lung, glioblastoma, ovarian cancers	$7.1 billion	Bevacizumab
Remicade	Rheumatoid arthritis, Crohn's disease, ankylosing spondylitis, psoriatic arthritis, plaque psoriasis, ulcerative colitis	$7.1 billion	Infliximab
Lantus	Diabetes	$5.7 billion	Insulin glargine
Neulasta	Cancer chemotherapy	$4.7 billion	Pegfilgrastim
Avonex	Multiple sclerosis	$2.1 billion	Interferon beta-1a
Lucentis	Age-related macular degeneration	$1.5 billion	Ranibizumab

HER2: human epidermal growth factor receptor 2.

a Blockbusters are defined here as products with annual sales of more than $1 billion.

The pattern of approval of biosimilars varies between countries, but there seem to have been two main ‘phases’ for this. The first phase occurred early on in the period following introduction and adoption of the biosimilar concept by the EU in 2006 with the CHMP approval of hormones and cytokines, e.g. somatropin (2006), erythropoietin (2007), and filgrastim (2008). The second phase occurred later (post 2012) with the approval of more product types and especially mAbs, e.g. infliximab (Republic of Korea, 2012; EU, 2013), trastuzumab (Republic of Korea, 2014), adalimumab (USA, 2016), rituximab (Republic of Korea, 2016) and bevacizumab (USA, 2017); see Fig. 2. The EU also approved numerous mAbs during this period and now has the largest number of approved mAb biosimilars (27 products) including 10 different adalimumab biosimilars; see Table 2.

It should be noted that not all products approved as biosimilars listed above and in the table are biosimilars as defined in the WHO, EU and USFDA guidelines. For example, the products listed under India and Iran may have been approved using other criteria. This may be the case for other products in the listings and the case for other products called ‘biosimilars’ by other countries which did not take part in the survey. Products called ‘biosimilars’ approved before 2006 are unlikely to be biosimilars (also see section 3.5).

Insulin and LMWH pose a problem for analysis here as they are regulated differently by different countries. In most countries taking part in the survey, insulin is regulated as a biological, but in the USA it is regulated as a chemical. The reasons for this are largely historical and administrative and the situation in some countries may change over time. This situation has allowed many biosimilar insulins (and insulin derivatives) to be approved in those countries which regulate insulin as a biological. However, this is not strictly possible if the substance is regulated as a chemical, where such products would presumably be regarded as generics. This situation is now more complex as the USFDA has made a change to the classification of insulin for biosimilar purposes as a biologic rather than a drug (chemical), which allows its approval of insulin biosimilars [15].

A similar situation exists for LMWH. Most countries regulate this as a biological, which implies that biosimilar LMWHs are approvable, which is the case in some countries e.g. Brazil and the EU. However, other countries continue to regulate these products as chemical drugs, e.g. Cuba, Iran, Japan, Republic of Korea, Russia. In these countries biosimilar LMWHs will not be approvable as a biological since there is no regulatory route for this. They can, presumably, be approved as generics using the chemical drugs regulatory pathway. It should be noted that WHO considers LMWHs to be biologicals based on their biological origin. Therefore, some of the guiding principles in the WHO biosimilar guidelines are applicable to LMWH.

### 3.4 Biosimilars under development

The success of biosimilars since their launch over 10 years ago has led to increasing investment in their development. Patent expiry has also allowed development of new biosimilar substances. Many biosimilars are claimed to be in development in many parts of the world; this seems to be a global trend. Past experience suggests that biosimilars produced by one country can be approved by other countries. In the past, certain ‘global players’ in the pharmaceutical industry dominated the market and were the major producers and/or licence/authorization holders. However more recently, many local manufacturers have developed biosimilars, e.g. in Egypt, India, Iran, Russia, Ukraine. If this trend continues, locally produced biosimilars may become the dominant products in some countries in the future. An unexpected finding in the survey is that about 20 biosimilars are under early stage development by local manufacturers in Egypt.

It is difficult to predict accurately which new products will be developed and approved due to the various uncertainties with this. The selection of products for development as biosimilars is influenced by several factors including patent expiry dates and the size of the sales of the originators (market penetration). A list of blockbusters is shown in Table 3. Most of these have biosimilar versions at present in some countries or are being planned for development as biosimilars.

The survey identified the following as being the prediction, based on products at present under development or planned. The situation in August 2019 with biosimilars under development is shown in Table 2.

The dominant product class for this is mAbs which are either immunosuppressants used for treatment of autoimmune disorders (e.g. Rheumatoid arthritis) or anticancer products, which reflects the recent experience with approved mAb biosimilars and their blockbuster status (see Table 2, Table 3).

The survey identified that at least 20 adalimumab, 25 bevacizumab, 23 rituximab and 20 trastuzumab biosimilar products are under development (preclinical and clinical development).

Other major biosimilar products being developed were etanercept (16 products) and pegfilgrastim (16 products). Some other products are in the pipeline, but generally in lower numbers e.g. pertuzumab, cetuximab and darbepoetin.

Biosimilar development seems to be generally expanding, although this needs to be followed carefully over time to identify progress and trends.

### 3.5 Quality of biosimilars

The quality of approved biosimilars is clearly crucial for their clinical safety and efficacy. Quality is normally ensured by requiring manufacturers to comply with all of the regulatory requirements for approval and release of biosimilar products. This includes post marketing surveillance and complying with good manufacturing practice (GMP).

Some products were licenced/approved in some countries prior to the adoption of biosimilar guidelines and/or the establishment of a regulatory framework for biosimilar approval. Consequently, there has been some public concern with the quality of biosimilars (and biologicals in general), although this does not seem to always be linked to direct evidence [[16], [17], [18]].

Russia had 17 products licensed prior to adoption of its guidelines in 2014; many of these were insulins from various sources. India approved more than 30 products during the period 2001–2011 prior to adoption of guidelines in 2012. Indonesia approved 14 products between 2001 and 2014. Before their own guidelines for biosimilar evaluation were issued in 2015, they used the EU guidelines (in 2005–2009) and WHO guidelines (after 2009) as the basis for approval of some of these products. Iran approved 11 products during the period from 1998 to 2013 although guidelines were not adopted until 2014. Some other countries e.g. Jordan and Ukraine also approved 6 and 2 biosimilars respectively before national guidelines were adopted but they used the EU (CHMP) guidelines as the basis for approval of these products.

The 14th International Conference of Drug Regulatory Authorities (ICDRA) discussed situations where, for various reasons, biotherapeutic products were licensed with data packages that do not follow current international regulatory standards for these biologicals [16]. This includes, for example, biotherapeutic products licensed via a generic pathway or with limited analytical, nonclinical and/or clinical data. The ICDRA requested WHO assistance in developing approaches for evaluating these already-licensed products, and WHO published a guidance document on a stepwise regulatory assessment of approved biotherapeutic products in 2015 [17]. As stated in the document, regulatory authorities need to reassess the products already approved under the pre-existing regulations to ensure that they meet the new requirements. In addition, it is important to note that biotherapeutics that have not been shown to be similar to a reference biotherapeutic product through the use of a head-to-head demonstration should neither be described as “similar” nor called biosimilars.

In an attempt to assist Member States which lack capacity or expertise to assess the quality of biosimilars, WHO has launched a pilot prequalification program for biosimilars used for cancer treatment [19]. For this, WHO is inviting manufacturers to submit applications for prequalification of two biotherapeutic products in the WHO Essential Medicines List: rituximab (used principally to treat non-Hodgkin's lymphoma and chronic lymphocytic leukaemia), and trastuzumab (used to treat breast cancer) and their corresponding SBPs. The aim of this is to make sure that the selected biosimilars are of good quality and safe, which is why prequalification will be a key step to expanding access to these medicines. WHO also intends to explore options for prequalifying biosimilar insulins.

## 4 Discussion and conclusions

There has been much progress in drafting and adopting guidance documents for biosimilars. All participating countries (and the USA) now have biosimilar guidelines in place and this has probably prompted the increased rate of approval of biosimilar products worldwide. As most of countries in the survey adopted WHO Guidelines for biosimilars, it is clear that WHO Guidelines have contributed to setting the regulatory framework for biosimilars in these countries and increasing regulatory convergence at the global level.

In some countries included in this assessment, all products called biosimilars comply with the definition of such products in the WHO Guidelines while in other countries products approved through different pathways may still be on the market. In most cases the number of approved biosimilars seems as expected, but China appears to be different from the rest in apparently having only 1 approved biosimilar. For such a large country, with a developed biotechnology industry this seems unusual, but is possibly explained by there being far more approved products which are ‘non-innovators’ rather than biosimilars in China. A difficulty in analyzing and particularly assessing the survey is being able to clearly distinguish between biosimilars and non-innovator products which have not been produced and evaluated according to the requirements of the WHO Guidelines [1]. This problem also existed at the time of the previous survey [11]. The coexistence on the market of these non-innovator products and biosimilars, as well as biotherapeutics licensed with full data packages, is a matter of concern. Non-innovator products already approved before biosimilar regulations exist may need to be reassessed by regulatory authorities and the terminology used for such products should not be confused by calling them biosimilars [17,18].

Nomenclature for biosimilars was noted as problematic in the report of the previous survey [11]. One of the achievements that WHO made with regulators in its Member States is that the term ‘biogeneric’ which was in use at the time of the first survey, seems to have been largely abandoned. The term ‘generic’ is not appropriate for any type of biologicals. WHO recommended avoiding use of the term SBP or biosimilar for products that have not been evaluated in line with WHO guiding principles for these products [1,16,17]. Although some attempt has been made to resolve the problem by using terminology that corresponds with the type of evaluation that a product has been subjected to, it is still an issue that requires additional effort to resolve and improve. Due to inappropriate and misleading use of the terms, it is still difficult to understand what is meant by the term ‘biosimilar’ in some countries. The inappropriate labelling of products as biosimilars is a barrier to the uptake of biosimilars as it decreases confidence in biosimilar use [18].

Biosimilars are now clearly established as safe and efficacious products in many counties and regions. A range of biosimilars is now available in all of the countries participating in the survey (and the USA), although the number of these varies. Biosimilars are being approved with increasing frequency and the competition within each product type is realizing the promise that adoption of biosimilars will reduce product costs and allow cheaper healthcare and greater patient access to important medicinal products [18]. This is particularly important for relatively expensive products, such as mAbs and the relatively large number of approved biosimilar mAb products is encouraging from this perspective.

The success with approval of biosimilars during the survey period and their fairly widespread use has prompted manufacturers to continue or expand their involvement with biosimilars and there are many products under development. These are both more of the existing repertoire of products and also new ones. The blockbuster status of some mAbs has led to a significant number of these being developed as biosimilars and this trend continues and is likely to continue in the future. In the past certain ‘global players’ in the pharmaceutical industry dominated the market and were the major producers and/or licence/authorization holders. However more recently many local manufacturers have developed biosimilars. If this trend continues, locally produced biosimilars may become the dominant products in some countries in the future. It is expected that the access to these products will increase at the global level and will contribute to the overall WHO goal highlighted in the World Health Assembly resolution in 2014 [20].

## Disclaimer

The authors alone are responsible for the views expressed in this article and they do not necessarily represent the views, decisions or policies of the institutions with which they are affiliated.

## Funding

10.13039/501100003625The Ministry of Health and Welfare, Republic of Korea provided the fund to WHO for this project through a voluntary contribution for the period of 1 December 2018 – 30 September 2019.

## Declaration of competing interest

The authors have disclosed no potential conflicts of interests.
